# Potential association of bone mineral density loss with cognitive impairment and central and peripheral amyloid-β changes: a cross-sectional study

**DOI:** 10.1186/s12891-022-05580-7

**Published:** 2022-06-30

**Authors:** Peng Zhang, Yi Zhou, Gang Chen, Jun Li, Bangjun Wang, Xinyan Lu

**Affiliations:** Department of Orthopedics, Xiangyang Central Hospital, Affiliated Hospital of Hubei University of Arts and Science, No. 136 Jingzhou Street, Xiangcheng District, Xiangyang, 441021 China

**Keywords:** Alzheimer’s disease, Bone mineral density, Cognitive impairment, Amyloid-β

## Abstract

**Background:**

There is some evidence in the literature that older adults with cognitive impairments have a higher risk for falls and osteoporotic hip fractures. Currently, the associations between bone health and cognitive health have not been extensively studied. Thus, the present cross-sectional study aims to investigate the relationship between markers of bone loss and cognitive performance in older adults with and without osteopenia as well as older adults with cognitive impairments (i.e., Alzheimer’s disease [AD]).

**Methods:**

Sixty-two non-osteopenia participants and one hundred three osteopenia participants as the cohort 1 and 33 cognitively normal non-AD participants and 39 AD participants as the cohort 2 were recruited. To assess cognitive and bone health, hip bone mineral density (BMD) and cognitive performance (via Minimal Mental State Examination [MMSE] and/or Auditory Verbal Learning Test-delayed recall [AVLT-DR]) were assessed. Furthermore, in cohort 1, plasma amyloid-β (Aβ) levels, and in cohort 2, cerebrospinal fluid (CSF) Aβ levels were determined.

**Results:**

We observed that (1) compared with non-osteopenia participants, BMD values (t = − 22.806; 95%CI: − 1.801, − 1.484; *p* < 0.001), MMSE scores (t = − 5.392; 95%CI: − 3.260, − 1.698; *p* < 0.001), and AVLT-DR scores (t = − 4.142; 95%CI: − 2.181, − 0.804; *p* < 0.001), plasma Aβ42 levels (t = − 2.821; 95%CI: − 1.737, − 0.305; *p* = 0.01), and Aβ42/40 ratio (t = − 2.020; 95%CI: − 0.009, − 0.001; *p* = 0.04) were significantly lower in osteopenia participants; (2) plasma Aβ42/40 ratio showed a mediate effect for the association between BMD values and the performance of cognitive function in osteopenia participants by mediation analysis, adjusting age, sex, years of education, and body mass index (BMI); (3) BMD values (95%CI: − 1.085, 0.478; *p* < 0.001) were significantly reduced in AD participants as compared with cognitively normal non-AD participants; (4) in AD participants, the interactive effects of BMD and CSF Aβ42/40 ratio on MMSE scores was found by regression analysis, controlling age, sex, years of education, and BMI; (5) BMD can distinguish AD participants from cognitively normal non-AD participants with AUC of 0.816 and distinguish participants with the cognitive impairment from cognitively normal participants with AUC of 0.794.

**Conclusion:**

Our findings suggest a relationship between bone health and cognitive health. Given the correlations between BMD and important markers of cognitive health (e.g.*,* central and peripheral pathological change of Aβ), BMD might serve as a promising and easy-accessible biomarker. However, more research is needed to further substantiate our findings.

## Background

Alzheimer’s disease (AD) is the most common cause of dementia, and with the population ages, the prevalence of AD is rising dramatically in the world [1]. The main pathologies of AD is the aberrant accumulation of amyloid-β (Aβ) peptides and neurofibrillary tangles (NFT) [2, 3]. The hallmark clinical characteristic of AD is progressive cognitive decline, especially episodic memory decline [4, 5]. To date, the precise diagnose of AD mainly depends on the cerebrospinal fluid (CSF) biomarkers (e.g. Aβ) and molecular positron emission tomography (PET) imaging [6, 7]. However, these biomarkers are difficult to assess in community screening due to their high invasiveness and costs. Furthermore, worse physical health is also an important issue in AD, such as weight loss, lower aerobic capacity, and motor dysfunction [8, 9].

Osteoporosis is a progressive skeletal disorder characterized by low bone mineral density (BMD) and impaired bone strength that can obviously increase the incidence of fracture [10, 11]. Epidemiological evidence supports increased bone loss are associated with impairment of cognitive function that may result in an increased risk of AD [12–15]. Currently, it is not fully clear whether osteoporosis or osteopenia, an early stage of osteoporosis, is a risk factor for AD or a complication caused by AD. However, in vitro and in vivo studies reveal that some relevant pathophysiological links may mediate the relationship between these two diseases, such as skeletal amyloid deposition and Vitamin D deficiency [16, 17]. Recently, the concept of “bone-brain crosstalk” has been introduced in the literature and postulate potential neurobiological pathways that linking bone health to cognitive health [18, 19]. In particular, it is hypothesized that the “bone-brain crosstalk” relies on the secretion of some osteoblast-derived molecules from the bone to the blood that, in turn, can influence the brain development and normal function of the central nervous system if they pass the blood-brain barrier [18, 19]. In addition, previous studies provide evidence that a link between BMD values (being measured by dual-energy X-ray absorptiometry [DXA]) and whole-brain volume (being measured by magnetic resonance imaging in AD) exists [20, 21], suggesting that bone loss may play an important role in the AD-related neurodegeneration. Hence, abnormal BMD may be a potential indicator to identify participants with a high risk of AD.

Plasma Aβ42 levels and Aβ42/40 ratio are strongly related to those of CSF and the degree of amyloid deposition in the brain [22–24], which suggests that plasma Aβ-related indicators may be reliable biomarkers to reflect central pathological changes in AD. In fact, the mRNA and protein expression levels of Aβ42 are increased significantly in the bone tissues in osteoporotic patients, and increased Aβ42 levels could aggravate the differentiation and activation of osteoclasts, which may be implicated in the pathogenesis of osteoporosis [25]. Furthermore, AD mouse models further indicate that Aβ is higher expressed in bone tissue and is correlated with the decreased BMD [26]. However, it is currently not fully clear whether bone health plays a crucial role in the development of cognitive decline since there is, to the best of our knowledge, no study available that investigate the possible relationship between markers of bone health (i.e.*,* BMD) and markers of cognitive health (i.e.*,* levels of Aβ in CSF) in clinical samples such as AD.

To address this gap in the literature, in the current study the associations between markers of bone health and cognitive health were investigated. In particular, we studied possible links between BMD and cognitive performance (assessed via Minimal Mental State Examination [MMSE] and Auditory Verbal Learning Test-delayed recall [AVLT-DR]) and between BMD and CSF/plasma Aβ levels. Furthermore, we also assessed the diagnostic value of BMD for AD or cognitive decline.

## Methods

### Participants

The present study was a cross-sectional study and included two independent cohorts.

#### Cohort 1

Sixty-two non-osteopenia participants and one hundred three osteopenia participants were recruited from Xiangyang Central Hospital. Each participant underwent a standardized interview for obtaining clinical information on the demographic characteristics, medical history, and physical and mental status. Furthermore, all participants performed the BMD measurement and collection of peripheral venous blood.

#### Cohort 2

Thirty-three cognitively normal non-AD participants and thirty-nine AD participants were also recruited from Xiangyang Central Hospital. A standardized interview for obtaining clinical information was also performed for all participants. Consistent standardized interview and the lumbar puncture were conducted for all participants. Cognitively normal non-AD participants and AD participants were defined according to the measurement of CSF Aβ40 and Aβ42, and diagnostic criteria are as follows: CSF Aβ42/Aβ40 ratio > 0.05 is normal and CSF Aβ42/Aβ40 ratio ≤ 0.05 is AD [27, 28].

All personal information of participants were obtained by self-reporting. Inclusion criteria for two cohorts were as follows: (1) age between 60 and 80 years old; (2) education year ≥8; (3) right-handed; (4) normal vision and hearing; (5) all participants had never taken any drugs that may affect the cognitive function, such as memantine, donepezil. In addition, individuals were excluded if they had: (1) a history of brain trauma; (2) neurological diseases that may affect the cognitive function (e.g. cerebrovascular disorders, Parkinson’s disease, multiple sclerosis); (3) any psychiatric disorders (e.g. major depressive disorder; schizophrenia); (4) other serious health problems (e.g., cancer, or impaired function of the liver or kidneys).

The present study obtained approval from the ethics committees of Xiangyang Central Hospital (approval ID: XYCH2018-018), and all participants signed written informed consent.

### Evaluation of cognitive function

For each participant of the cohort 1, the global cognitive function of participants were assessed using MMSE scale [29] which is an effective screening tool for dementia and assess the ability of orientation to time and place, memory, computational power, information processing speed, executive function, visual-spatial ability and language ability, and episodic memory function were assessed using AVLT-DR scale (the time of delayed recall is 20 min) [30] due to the episodic memory is characteristically impaired during AD [31, 32]. However, for the cohort 2, only MMSE assessment was performed.

### BMD measurement

BMD (g/cm^2^) of each participant was measured using DEXA (Dexa Pro-II, Pinyuan Electronic Technology Co., Ltd., Xuzhou, China). According to the number of standard deviations providing by the manufacturer, the T-score of participant was determined. Health, osteopenia, or osteoporosis was defined according to the measured BMD, represented by T-score of the hip [11, 33]. Diagnostic criteria are as follows: T-score ≥ − 1.0 is healthy, − 2.5 < T-score < − 1 is osteopenia, and T-score ≤ − 2.5 is osteoporosis.

### The measurement of CSF AD-related indicators

For the cohort 2, about 10 ml of CSF was collected in polypropylene tubes and was immediately centrifuged for 10 min at 2000 g at 4 °C within 2 h. Then, samples were aliquoted and stored at − 80 °C.

The concentrations of CSF Aβ40 and Aβ42 were measured using sandwich enzyme-linked immunosorbent assays (ELISAs) (INNOTEST, Fujirebio, Belgium) according to the kit instructions. CSF samples belonging to the same participants were measured in triplicate with the same standard. The samples would be measured again, if intra-assay coefficients of variation > 11.0%.

### The measurement of plasma AD-related indicators

For the cohort 1, peripheral venous blood samples were collected after overnight fasting between 8:00 AM and 9:00 AM. Afterward, the blood samples were centrifuged at 1500 g for 10 minutes at 4 °C to obtain blood plasma. Then, the samples were aliquoted and stored at − 80 °C.

The concentration of plasma Aβ40 and Aβ42 were measured in triplicate using the Quanterix Simoa-HD1 Platform (Simoa; Quanterix, Lexington, MA, USA) [34, 35], and Neurology 3-Plex A kits were used according to the kit protocol. The intra-assay and inter-assay variability were below 12%.

### Statistical analysis

All data analyses were performed using SPSS version 22.0 (SPSS, Inc., Chicago, IL, USA). The Kolmogorov-Smirnov test was used to assess the normal distribution of the data and the Levene’s test was used to verify the presence or absence of variance homogeneity. Categorical variable (i.e. sex) was analyzed using a chi-squared test, and continuous variables (i.e. age, years of education, body mass index [BMI], BMD, cognitive assessments, and CSF/plasma AD-related indicators’ levels) were analyzed using an independent-sample t-test. Effect size was computed using Cohen’s d. Correlation analysis [36, 37] and linear regression analysis were used to investigate for a possible relationship between BMD and cognitive function and CSF/plasma AD-related indicators, and all analyses were performed with age, sex, years of education, and BMI as covariates. Statistical significance was set at *P* < 0.05 (two tailed).

Furthermore, given the potential influence of AD-related indicator and BMD on the cognitive impairment in participants, we performed mediation analysis to further determine whether AD-related indicator could mediate the association between BMD and cognitive impairment, which is based on a standard three-variable mediation model [38, 39]. Covariates included age, sex, education years, and BMI. In the present analysis, three steps regression models were build, as shown below:

1 $$Y= cX+e1$$

2 $$M= aX+e2$$

3 $$\mathrm{Y}={c}^{'}\mathrm{X}+b\mathrm{M}+e3\kern0.5em \mathrm{or}\kern0.5em z=\frac{ab}{\sqrt{\left({b}^2{\mathrm{SE}}_a^2\right)-\left({a}^2{\mathrm{SE}}_b^2\right)}}$$

Where X is the dependent variable (BMD), Y is the independent variable (cognitive assessments), M is the mediator (plasma Aβ42/40 ratio), a is the regression coefficient for the relationship between BMD and plasma Aβ42/40 ratio, b is the regression coefficient for the relationship between plasma Aβ42/40 ratio and cognitive assessments, c is the regression coefficient for the relationship between BMD on cognitive assessments. z and c’ represent the effect of BMD on cognitive assessments when controlling for the indirect effect. SEa is the standard error of the relationship between BMD and plasma Aβ42/40 ratio, and SEb is the standard error of the relationship between plasma Aβ42/40 ratio and cognitive assessments. Then, mediation effect are defined based on following 4 conditions: (1) c must be significant; (2) a and b are significant; (3) |c’| < |c| (partial mediation) or c’ is insignificant (full mediation); (4) if a or b is insignificant, z must be significant. We used ratio indirect to present the strength of mediation ([a*b]/c) if there is mediation effect in the present analysis.

In addition, receiver operator characteristic (ROC) curve analysis was used to compute the area under the curve (AUC) to determine the diagnostic value of BMD for the cognitive impairment (or AD). The Youden index was used to obtain optimal sensitivity and specificity.

## Results

### Analysis of the cohort 1 (non-osteopenia individuals and osteopenia individuals)

#### Comparison of demographic variables, cognitive function, and plasma levels of AD-related indicators

There are no between-group differences concerning demographic parameters (i.e.*,* age, sex, years of education, BMI; Table 1), but BMD values (t = − 22.806; 95%CI: − 1.801, − 1.484; *p* < 0.001), MMSE scores (t = − 5.392; 95%CI: − 3.260, − 1.698; *p* < 0.001) and AVLT-DR scores (t = − 4.142; 95%CI: − 2.181, − 0.804; *p* < 0.001) were lower in older adults with osteopenia as compared to non-osteopenia participants (Table 1). Additionally, compared with non-osteopenia participants, plasma Aβ42 levels (t = − 2.821; 95%CI: − 1.737, − 0.305; *p* = 0.01) and Aβ42/40 ratio (t = − 2.020; 95%CI: − 0.009, − 0.001; *p* = 0.04) were significantly lower in participants with osteopenia (Table 1). However, there were no significant difference in plasma Aβ40 levels between the two groups (Table 1).

**Table 1:** Comparison of demographic variables, BMD, cognitive function assessments, and plasma AD-related indicators in the cohort 1

	Non-osteopenia (*n* = 62)	Osteopenia (*n* = 103)	95% CI	t /χ^2^ value	*P*-value	Effect size	Power
Age (years)	67.24 ± 5.46	66.62 ± 5.63	−2.378, 1137	−0.693	0.49^a^	0.11	0.10
Sex (M / F)	29 / 33	39 / 64	–	1.268	0.26^b^	–	–
Education (years)	11.21 ± 3.04	11.30 ± 2.63	−0.794, 0.977	0.204	0.84^a^	0.03	0.05
BMI	23.13 ± 3.67	22.71 ± 3.34	−1.516, 0.685	−0.746	0.46^a^	0.12	0.11
BMD (g/cm^2^)	0.17 ± 0.56	−1.48 ± 0.37	−1.801, − 1.484	−22.806	< 0.001^a^	3.48	0.99
MMSE scores	28.03 ± 1.69	25.55 ± 3.37	−3.260, − 1.698	−5.392	< 0.001^a^	0.93	0.99
AVLT-DR scores	5.98 ± 2.99	4.33 ± 2.12	−2.181, − 0.804	−4.142	< 0.001^a^	0.64	0.96
Plasma Aβ40 (pg/ml)	236.67 ± 59.53	231.72 ± 39.38	− 24.702, 9.365	− 0.894	0.37^a^	0.10	0.10
Plasma Aβ42 (pg/ml)	14.57 ± 1.00	13.55 ± 3.44	−1.737, −0.305	− 2.821	0.01^a^	0.42	0.70
Plasma Aβ42/Aβ40 ratio	0.07 ± 0.01	0.06 ± 0.02	−0.009, − 0.001	−2.020	0.04^a^	0.63	0.95

*Abbreviations*: *CI* Confidence intervals, *BMI* Body mass index, *BMD* Bone mineral density, *M / F* Male / female, *MMSE* Mini-Mental State Examination, *AVLT-20 min DR* Auditory Verbal Learning Test-20-minute delayed recall, *Aβ* Amyloid-β

Data are presented as the mean ± stand deviation

^a^*p*-values were obtained by independent-sample t-test; ^b^*p*-values were obtained by χ^2^ test

#### Relationship of BMD values with cognitive assessments and plasma AD-related indicators

**Fig. 1:**
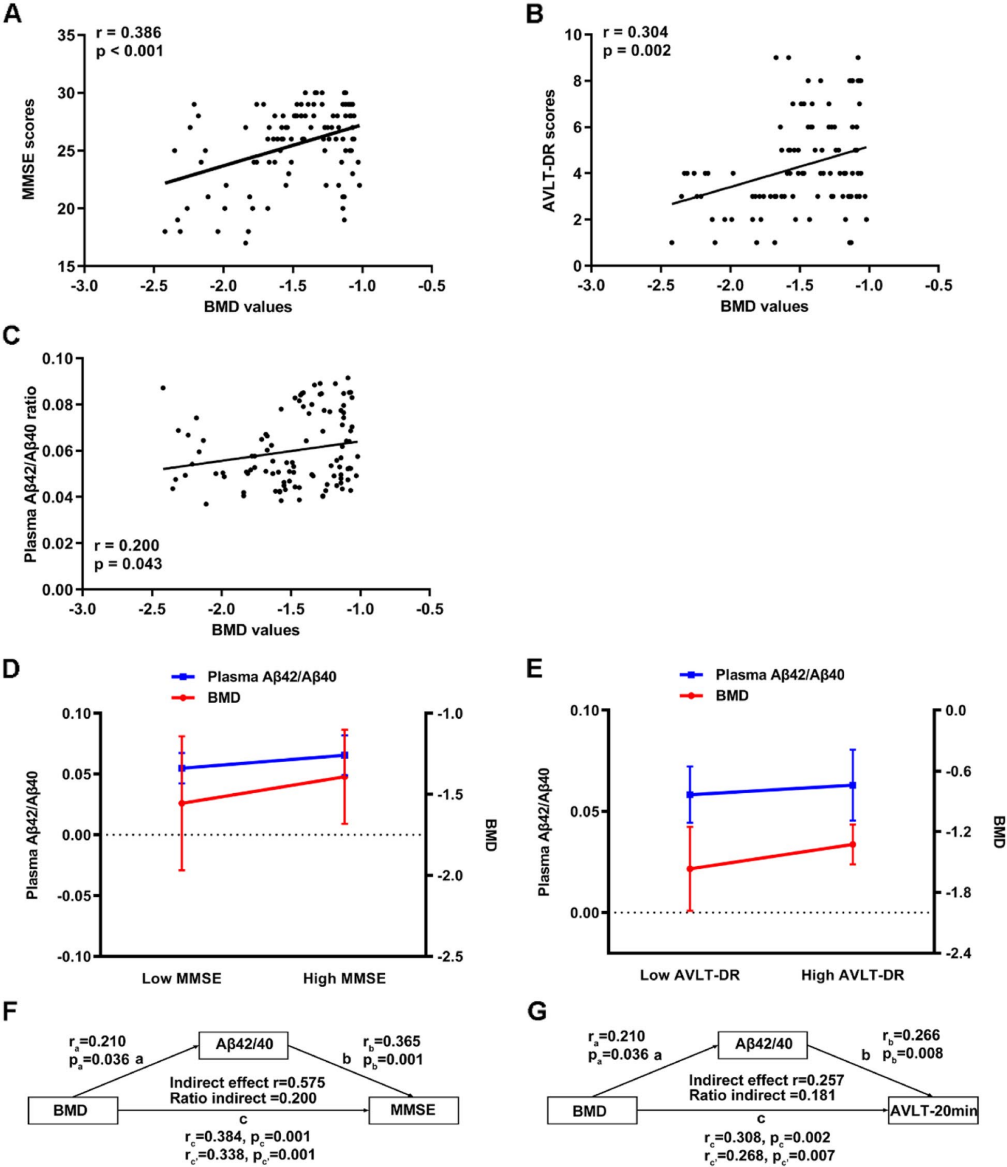
Analyses of the association between BMD and cognition and plasma Aβ42/Aβ40 in individuals with osteopenia in the cohort 1. **A** Correlation analysis between BMD values and MMSE scores. **B** Correlation analysis between BMD values and AVLT-DR scores. **C** Correlation analysis between BMD values and plasma Aβ42/Aβ40 ratio. **D** Interactive effect of BMD and plasma Aβ42/Aβ40 ratio on MMSE scores. **E** Interactive effect of BMD and plasma Aβ42/Aβ40 ratio on AVLT-DR scores. **F** Mediation effects of plasma Aβ42/Aβ40 ratio on the association between BMD and MMSE scores in participants with osteopenia. **G** Mediation effects of plasma Aβ42/Aβ40 ratio on the association between BMD and AVLT-DR scores. Abbreviations: Aβ, amyloid-β; BMD, bone mineral density; MMSE, Mini-Mental State Examination; AVLT-DR, Auditory Verbal Learning Test-delayed recall

In participants with osteopenia, there were positive correlations between BMD values and cognitive assessment scores (MMSE: r = 0.386, *p* < 0.001, Fig. 1A; AVLT-DR: r = 0.304, *p* = 0.002, Fig. 1B). Furthermore, the higher plasma Aβ42/Aβ40 ratio were positively correlated with the higher BMD values participants with osteopenia (r = 0.200, *p* = 0.043, Fig. 1C).

The regression analysis further indicated that in participants with osteopenia, lower BMD values and plasma Aβ42/40 ratio were significantly associated with reduced MMSE scores and AVLT-DR scores (Table 2), which indicated that the interactive effects of BMD values and plasma Aβ42/40 ratio on the MMSE or AVLT-DR scores occurred in osteopenia participants. In order to depict the specific interactive pattern and the results of regression analysis, participants were divided into low and high MMSE/AVLT-DR based on the means values, which can be found in previous study [39]. We divided participants into a “low MMSE” group (53 participants with MMSE scores ≤26) and a “high MMSE” group (50 participants with MMSE scores > 26). Participants with lower MMSE scores showed lower BMD values and lower plasma Aβ42/40 ratio (Fig. 1D). Besides, participants with higher AVLT-DR scores (64 participants with AVLT-DR scores ≤4) had higher BMD values and higher plasma Aβ42/40 ratio, while participants with lower AVLT-DR scores (39 participants with AVLT-DR scores > 4) had lower BMD values and lower plasma Aβ42/40 ratio (Fig. 1E).

**Table 2:** The regression analysis of cognitive assessments in individuals with osteopenia in the cohort 1

	MMSE scores		AVLT-DR scores	
Age (years)	β = 0.064	*P* = 0.091	β = − 0.011	*P* = 0.908
Sex (M / F)	β = − 0.060	*P* = 0.502	β = 0.019	*P* = 0.839
Education (years)	β = 0.006	*P* = 0.949	β = − 0.037	*P* = 0.706
BMI	β = 0.038	*P* = 0.675	β = 0.021	*P* = 0.829
BMD (g/cm^2^)	β = 0.320	*P* = 0.001	β = 0.264	*P* = 0.008
Plasma Aβ42/Aβ40 ratio	β =0.303	P = 0.001	β = 0.214	*P* = 0.032

*Abbreviations*: *BMI* Body mass index, *BMD* Bone mineral density, *M / F* Male / female, *MMSE* Mini-Mental State Examination, *AVLT-20 min DR* Auditory Verbal Learning Test-20-minute delayed recall, *Aβ* Amyloid-β

The results indicated two independent regression models. Model 1: Dependent variable = MMSE scores; Independent variable = Age, Sex, Education, BMI, BMD, and Plasma Aβ42/Aβ40 ratio. Model 2: Dependent variable = AVLT-DR scores; Independent variable = Age, Sex, Education, BMI, BMD, and Plasma Aβ42/Aβ40 ratio

Mediation analysis further indicated that in the osteopenia group, plasma Aβ42/40 ratio acted as a mediator between BMD values and MMSE and AVLT-DR assessment, respectively, adjusting age, sex, education years, and BMI (Fig. 1F and G).

### Analysis of the cohort 2 (cognitively normal non-AD individuals and AD individuals)

#### Comparison of demographic variables, cognitive function, and CSF levels of AD-related indicators

Compared with the cognitively normal non-AD participants, BMD values (t = − 5.137; 95%CI: − 1.085, 0.478; *p* < 0.001), MMSE scores (t = − 15.487; 95%CI: − 14.287, − 11.027; *p* < 0.001), CSF Aβ40 levels (t = − 2.201; 95%CI: 127,023, 2576.743; *p* = 0.03), Aβ42 levels (t = − 6.284; 95%CI: − 275.293, − 141.924; *p* < 0.001) and CSF Aβ42/40 ratio (t = − 15.223; 95%CI: − 0.030, − 0.023; *p* < 0.001) were significantly reduced in the AD group (Table 3). However, other clinical features including age, sex, education years, and BMI, had no significant difference between the two groups (Table 3).

**Table 3:** Comparison of demographic variables, BMD, cognitive function assessment, and CSF AD-related indicators’ levels in the cohort 2

	Non-AD (*n* = 33)	AD (*n* = 39)	95% CI	t /χ^2^ value	*P*-value	Effect size	Power
Age (years)	68.70 ± 6.50	70.74 ± 5.32	−0.731, 4.824	1.470	0.15^a^	0.34	0.28
Sex (M / F)	18 / 21	16 / 17	–	0.039	0.84^b^	–	–
Education (years)	10.85 ± 2.51	11.27 ± 3.01	−0.896, 1.738	0.637	0.53^a^	0.15	0.09
BMI	22.33 ± 3.27	23.43 ± 3.37	−0.473, 2.667	1.393	0.17^a^	0.33	0.27
BMD (g/cm^2^)	−1.01 ± 0.72	−1.79 ± 0.57	− 1.085, 0.478	− 5.137	< 0.001^a^	1.20	0.99
MMSE scores	25.27 ± 3.44	12.62 ± 3.47	− 14.287, − 11.027	−15.487	< 0.001^a^	3.66	0.99
CSF Aβ40 (pg/ml)	11,114.57 ± 2698.62	9762.69 ± 2469.78	127,023, 2576.743	−2.201	0.03^a^	0.52	0.69
CSF Aβ42 (pg/ml)	628.26 ± 167.72	419.65 ± 98.68	−275.293, − 141.924	−6.284	< 0.001^a^	1.52	0.99
CSF Aβ42/Aβ40 ratio	0.07 ± 0.01	0.04 ± 0.01	−0.030, − 0.023	−15.223	< 0.001^a^	3.00	0.99

*Abbreviations*: *AD* Alzheimer’s disease, *CI* Confidence intervals, *BMI* Body mass index, *BMD* Bone mineral density, *M / F* Male / female, *MMSE* Mini-Mental State Examination, *CSF* Cerebrospinal fluid

Data were presented as the mean ± stand deviation

^a^ *p*-values were obtained by independent-sample t-test; ^b^ *p*-values were obtained by χ^2^ test

### Relationship of BMD values with cognitive assessments and CSF AD-related indicators

**Fig. 2:**
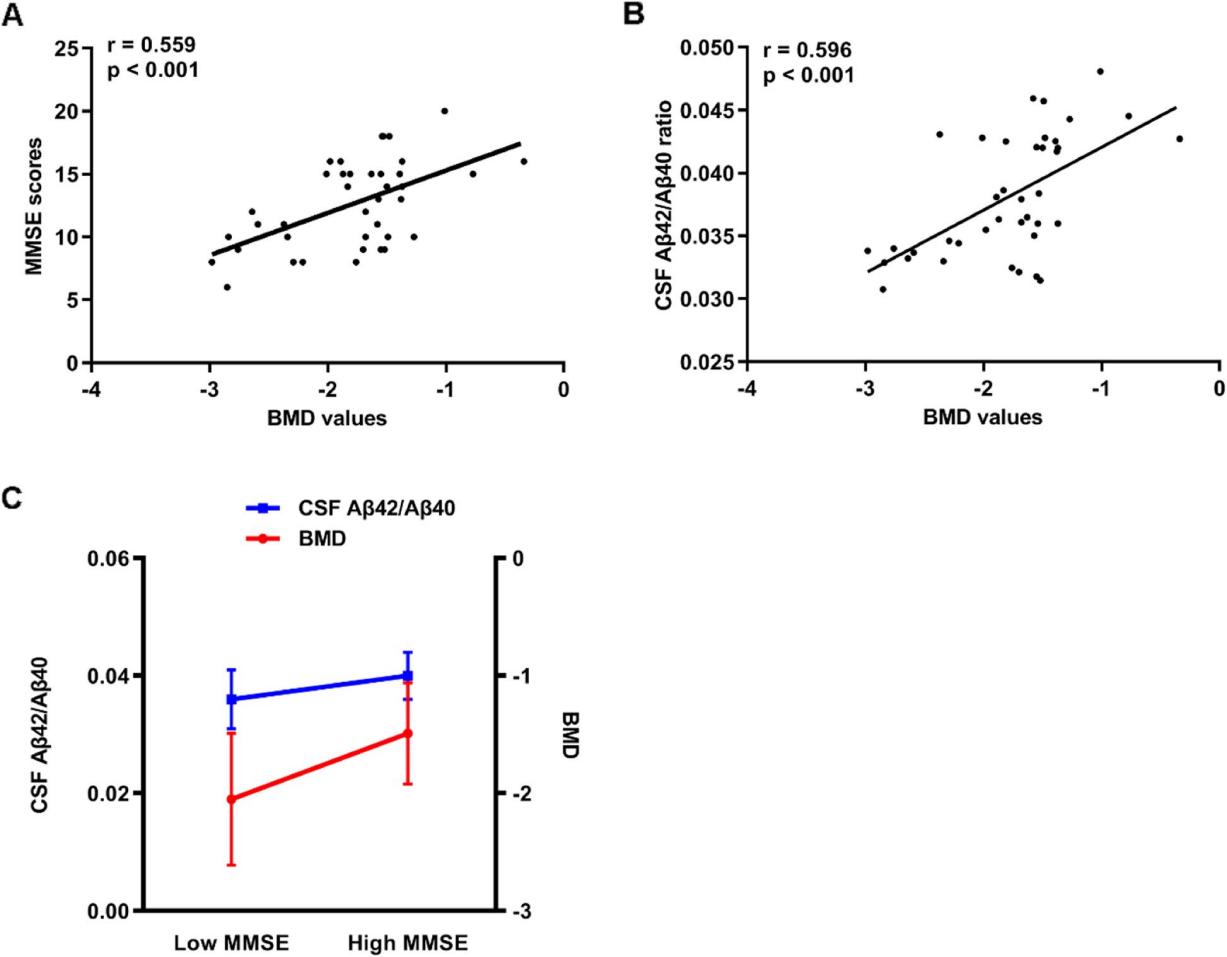
Analyses of the association between BMD and cognition and CSF Aβ42/Aβ40 ratio in AD individuals in the cohort 2. **A** Correlation analysis between BMD values and MMSE scores. **B** Correlation analysis between BMD values and CSF Aβ42/Aβ40 ratio. **C** Interactive effect of BMD and CSF Aβ42/Aβ40 ratio on MMSE scores. Abbreviations: CSF, cerebrospinal fluid; Aβ, amyloid-β; BMD, bone mineral density; AD, Alzheimer’s disease; MMSE, Mini-Mental State Examination

In AD participants, BMD values were positively correlated with MMSE scores (r = 0.559, *p* < 0.001, Fig. 2A) and CSF Aβ42/40 ratio (r = 0.596, *p* < 0.001, Fig. 2B). Furthermore, the interactive effects of BMD values and CSF Aβ42/40 ratio on the MMSE scores was also found in AD participants using the regression analysis (Table 4). To facilitate the exhibition of results of regression analysis, participants were divided into low and high MMSE based on the means values [39]. We divided participants into a “low MMSE” group (21 participants with MMSE scores ≤13) and a “high MMSE” group (18 participants with MMSE scores > 13). Participants with lower MMSE scores showed lower BMD values and lower CSF Aβ42/40 ratio (Fig. 2C).

**Table 4:** The regression analysis of the MMSE assessment in AD individuals in the cohort 2

	MMSE scores	
Age (years)	β = − 0.070	*P* = 0.665
Sex (M / F)	β = − 0.185	*P* = 0.231
Education (years)	β = − 0.068	*P* = 0.612
BMI	β = − 0.017	*P* = 0.902
BMD (g/cm^2^)	β = 0.368	*P* = 0.038
CSF Aβ42/Aβ40 ratio	β = 0.339	*P* = 0.047

*Abbreviations*: *BMI* Body mass index, *BMD* Bone mineral density, *M / F* Male / female, *MMSE* Mini-Mental State Examination, *CSF* Cerebrospinal fluid, *Aβ* Amyloid-β

The results displayed one regression model. Dependent variable = MMSE scores; Independent variable = Age, Sex, Education, BMI, BMD, and CSF Aβ42/Aβ40 ratio

### Diagnostic value of BMD for AD

**Fig. 3:**
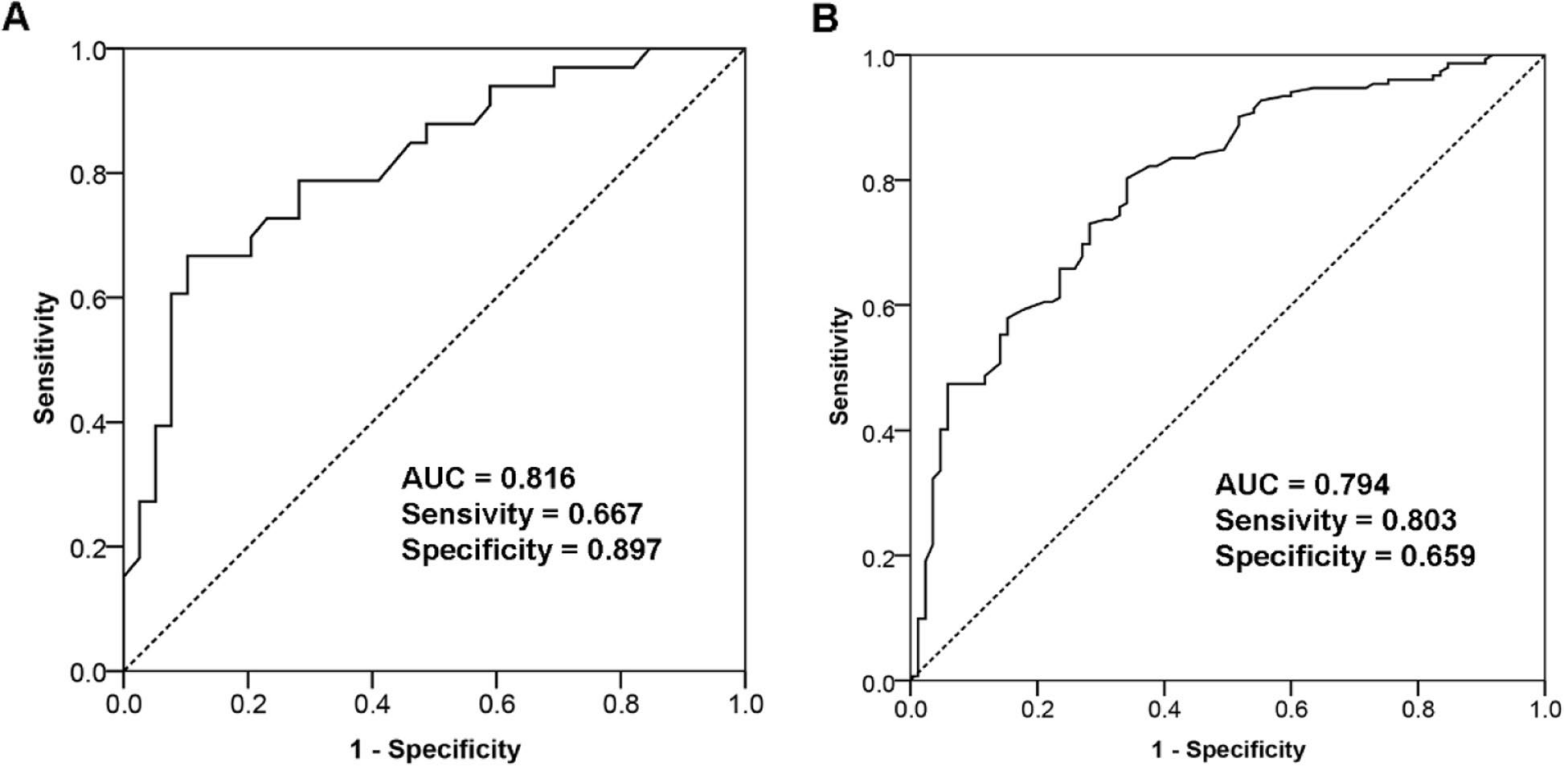
ROC curve analysis. **A** In the cohort 2, the diagnostic value of BMD between AD participants and cognitively normal non-AD participants. **B** In the combined cohort, the diagnostic value of BMD between participants with the cognitive impairment and cognitively normal participants. Abbreviations: ROC, receiver operator characteristic; BMD, bone mineral density; AD, Alzheimer’s disease

ROC curve indicated that BMD can distinguish AD participants from cognitively normal non-AD participants (AUC = 0.816; Fig. 3A). In addition, to further assess the diagnostic value of BMD for the cognitive impairment, we combined two cohorts for the ROC curve analysis and subsequently divided into two groups based on the MMSE scores (> 24 score and ≤ 24 score). We found that BMD exhibited an AUC value of 0.794 to classify participants with the cognitive impairment (*n* = 85) from participants with the normal cognition (Fig. 3B).

## Discussion

In this cross-sectional study, we found that (1) MMSE and AVLT-DR scores and plasma Aβ42 and Aβ42/40 ratio were significantly lower in participants with osteopenia as compared with non-osteopenia participants; (2) in participants with osteopenia, reduced BMD values were positively correlated with the poor MMSE and AVLT-DR scores and the lower plasma Aβ42/40 ratio, respectively, controlling age, sex, years of education, and BMI; (3) with adjusting age, sex, years of education, and BMI, plasma Aβ42/40 ratio could indirectly mediate the relationship between BMD and the performance of the cognitive function in osteopenia participants; (4) when compared with cognitively normal non-AD participants, BMD values, MMSE, and CSF Aβ42 levels and Aβ42/40 ratio were significantly reduced in AD participants; (5) in AD participants, BMD values were positively correlated with MMSE scores and CSF Aβ42/40 ratio, and the interaction between BMD and CSF Aβ42/40 ratio could affect MMSE scores, adjusting age, sex, years of education, and BMI; (6) BMD could differentiate participants with AD or cognitive impairment from cognitively normal participants. Taken together, these findings suggested that BMD may be a potential indicator for evaluating the performance of cognitive impairment, and aberrant central and peripheral change of Aβ may be an important factor to mediate the relationship between BMD values and the abnormal cognition.

In the present study, we firstly measured plasma Aβ levels using an ultrasensitive quantitative method and assessed the performance of episodic memory using the specific assessment scale in participants with osteopenia [40, 41]. Besides, we obtained the definitive diagnosis of AD based on the CSF Aβ levels according to the newest international diagnosis criterion [6, 7] and provided strong evidence on the association between BMD and central Aβ levels for the first time. These works of the present study would contribute to interpreting the associations between bone health and cognitive health.

In according with previous studies [12, 42, 43], the present study showed that the global cognitive function of osteopenia participants was reduced and low BMD values were significantly correlated with the poor global cognitive function, which suggested that osteopenia (lower BMD values) may be a higher risk of the cognitive decline. Furthermore, we further evaluated the performance of episodic memory (measured by the AVLT-DR scale) and detected that the reduced episodic memory was significantly associated with decreased BMD values, which suggested that the episodic memory may be the primary cognitive domain that is impaired in participants with osteoporosis. Additionally, we firstly detected a positive correlation between BMD values and plasma Aβ42/40 ratio, suggesting that BMD is a valuable indicator which may be related with the pathophysiology of amyloid-β. In addition, the present study found that the interactive effect of BMD and plasma Aβ42/40 ratio could reflect the performance of global cognitive function and episodic memory, and the association between BMD and cognitive function could be mediated by plasma Aβ42/40 ratio, which we speculate may indicated that abnormal bone health may affect the cognitive health through the Aβ-related pathology.

Although previous studies indicated that plasma Aβ42/40 ratio could closely correlate with the levels of AD-related cerebral pathological biomarkers (e.g.*,* amyloid deposition measured by positron emission tomography, CSF Aβ42/40 ratio) and plasma Aβ42/40 ratio could be regarded as an effective biomarker for assessing the state of Aβ cerebral deposition and cognitive dysfunction [44, 45], it is still an indirect evidence (blood-derived indicator) to assess the association between BMD and cerebral pathological change of AD. Therefore, we further evaluate the clinical value of BMD in AD participants with the measurement of CSF AD-related indicators. Consistent with previous studies [20, 46], we found that BMD was significantly reduced in AD participants when compared with cognitively normal non-AD participants. Furthermore, in the present study, the positive correlations of low BMD with reduced MMSE scores and low CSF Aβ42/40 ratio were detected in AD participants, and most importantly, we determined that the interaction between BMD and CSF Aβ42/40 ratio could result in the cognitive impairment, which further confirmed the finding of the cohort 1. Thus, we speculated that bone loss may affect the cognitive function via a central pathologic change of amyloid-β.

The present study detected that BMD could identify AD participants with high accuracy. Furthermore, for a combined and larger samples, BMD could also accurately distinguish participants with the cognitive impairment from cognitively normal participants, which indicated that low BMD may contribute to identifying AD patients or participants with cognitive decline.

The present study has certain limitations. (1) This study lacks the analysis of CSF AD-related pathological indicators in participants with osteopenia (the cohort 1), because of it is difficult to conduct a lumbar puncture in participants with the risk of fracture. (2) A larger sample size of patients with complete lumbar puncture is necessary to confirm the present findings in the future study. In the subsequent study, we will expand the sample size to confirm the present finding, including mild cognitive impairment patients, vascular dementia patients. Moreover, a longitudinal study is also necessary to further determine the order of early cognitive loss and bone loss due to the present study is only a cross-sectional study. (3) Important confounder such as physical fitness level, level of regular physical activity, or diet being known to influence bone health [47–49], are not assessed in this study. Thus, the findings of the current study should be interpreted in light of this limitation and future studies are needed to rule out whether these lifestyle factors influence the observed relationships between markers of bone health and markers of cognitive health.

## Conclusion

In the present study, we observed that in both older adults with osteopenia and older adults with AD as compared to cognitively normal participants, BMD values were lower and were associated with the severity of cognitive impairments. In addition, we provided preliminary evidence that the peripheral and central Aβ42/40 ratio mediate the relationship of BMD and severity of cognitive impairments in older adults with osteopenia and older adults with AD, respectively. Therefore, lower BMD values are not only a useful indicator reflecting a heightened risk of bone fractures but can also serve as a potential biomarker concerning cognitive health. However, future research investigating the link between bone health and cognitive health is needed to further substantiate the available evidence suggesting that both health domains are mutually linked.

## Data Availability

The datasets generated during and analyzed during the current study are not publicly available due to these data are being further analyzed by other members of our research team but are available from the corresponding author on reasonable request.
